# Glucocorticoid Withdrawal Syndrome following treatment of endogenous Cushing Syndrome

**DOI:** 10.1007/s11102-022-01218-y

**Published:** 2022-04-26

**Authors:** Xin He, James W. Findling, Richard J. Auchus

**Affiliations:** 1 Department of Internal Medicine, Division of Metabolism, Endocrinology and Diabetes, University of Michigan Medical School, Ann Arbor, MI USA; 2 Department of Medicine, Division of Endocrinology and Molecular Medicine, Medical College of Wisconsin, Milwaukee, WI USA; 3 Endocrinology Center and Clinics, Medical College of Wisconsin, Milwaukee, WI USA; 4 Department of Pharmacology, University of Michigan Medical School, Ann Arbor, MI USA; 5 Lieutenant Colonel Charles S. Kettles Veterans Affairs Ann Arbor Healthcare System, Ann Arbor, MI USA

**Keywords:** Glucocorticoid withdrawal syndrome, Steroid withdrawal syndrome, Corticosteroid withdrawal syndrome, Cushing syndrome, Cushing disease, Adrenal insufficiency

## Abstract

**Purpose:**

Literature regarding endogenous Cushing syndrome (CS) largely focuses on the challenges of diagnosis, subtyping, and treatment. The enigmatic phenomenon of glucocorticoid withdrawal syndrome (GWS), due to rapid reduction in cortisol exposure following treatment of CS, is less commonly discussed but also difficult to manage. We highlight the clinical approach to navigating patients from GWS and adrenal insufficiency to full hypothalamic-pituitary-adrenal (HPA) axis recovery.

**Methods:**

We review the literature on the pathogenesis of GWS and its clinical presentation. We provide strategies for glucocorticoid dosing and tapering, HPA axis testing, as well as pharmacotherapy and ancillary treatments for GWS symptom management.

**Results:**

GWS can be difficult to differentiate from adrenal insufficiency and CS recurrence, which complicates glucocorticoid dosing and tapering regimens. Monitoring for HPA axis recovery requires both clinical and biochemical assessments. The most important intervention is reassurance to patients that GWS symptoms portend a favorable prognosis of sustained remission from CS, and GWS typically resolves as the HPA axis recovers. GWS also occurs during medical management of CS, and gradual dose titration based primarily on symptoms is essential to maintain adherence and to eventually achieve disease control. Myopathy and neurocognitive dysfunction can be chronic complications of CS that do not completely recover.

**Conclusions:**

Due to limited data, no guidelines have been developed for management of GWS. Nevertheless, this article provides overarching themes derived from published literature plus expert opinion and experience. Future studies are needed to better understand the pathophysiology of GWS to guide more targeted and optimal treatments.

**Supplementary Information:**

The online version contains supplementary material available at 10.1007/s11102-022-01218-y.

## Introduction

Endogenous neoplastic hypercortisolism - Cushing syndrome (CS) - is one of the most challenging diagnostic and management problems in clinical endocrinology. CS may be due to either a pituitary tumor (Cushing disease, CD), or a non-pituitary (ectopic) tumor secreting ACTH. ACTH-independent hypercortisolism due to unilateral or bilateral adrenal nodular disease has been increasingly recognized as an important cause of CS. Regardless of the cause of CS, the clinical manifestations are protean and include a myriad of clinical, biochemical, neurocognitive, and neuropsychiatric abnormalities. The catabolic state of hypercortisolism causes signs and symptoms including skin fragility, bruising, delayed healing, violaceous striae, muscle weakness, and low bone mass with fragility fractures. Other clinical features include weight gain, fatigue, depression, difficulty concentrating, insomnia, facial plethora, and fat redistribution to the head and neck with resultant supraclavicular and dorsocervical fullness[1]. Metabolic consequences of hypercortisolism including hypertension, diabetes, and dyslipidemia are common. In addition, women often experience hirsutism and menstrual irregularity, while men may have hypogonadism.

Management options of CS include surgery, medications, and radiation. The preferred first line treatment, regardless of source, is surgery, which offers the potential for remission[2–4]. The primary literature, reviews, and clinical practice guidelines for CS have traditionally focused on the diagnosis, subtyping, and surgical approach to CS. This bias derives first from the profound diagnostic challenge posed in the evaluation of cortisol production and dynamics, given that circulating cortisol follows a circadian rhythm, exhibits extensive protein binding and metabolism, and rises acutely with stress. CD and ectopic ACTH syndrome may be difficult to distinguish clinically and biochemically, and inferior petrosal sinus sampling is required in many patients to resolve this differential diagnosis. Ectopic ACTH-producing tumors can also be small, and these tumors can escape localization despite the best current methods. Although diagnosis and initial surgical remission can be achieved in the majority of patient with CS at experienced centers, up to 50% of patients with CD will require additional therapies after unsuccessful primary surgeries or recurrence up to many years later[5]. For patients who do not achieve surgical cure or who are not surgical candidates, several medical treatment options are now available. Pharmacotherapies directed at the pituitary include pasireotide[6, 7] (FDA approved) and cabergoline[8]. Adrenal steroidogenesis inhibitors such as osilodrostat[9] (FDA approved), metyrapone[10], levoketoconazole[11] (FDA approved) and ketoconazole[12], as well as the glucocorticoid antagonist, mifepristone[13] (FDA approved), are now widely used to treat CS. Pituitary radiotherapy is an additional treatment option for CD but can take months to years to lower cortisol production. Bilateral adrenalectomy (BLA) provides immediate, reliable correction of hypercortisolism but mandates life-long corticosteroid replacement therapy, and, in patients with CD, may be complicated by corticotroph tumor progression syndrome in 25–40% of patients[14].

After successful surgery for CS, the rapid onset of adrenal insufficiency (AI) is anticipated and usually portends a favorable prognosis [15–18]; however, despite the use of post-operative corticosteroid replacement, the rapid reduction in cortisol exposure often results in an enigmatic phenomenon referred to as the glucocorticoid withdrawal syndrome (GWS). This article addresses the clinical presentation and the pathogenesis of GWS, as well as its distinction from AI. When available, appropriate references are provided. Statements and guidance provided without references are derived from expert opinion and experience.

## Clinical Presentation and Pathogenesis of GWS

GWS occurs following withdrawal of supraphysiologic exposure to either exogenous or endogenous glucocorticoids of at least several months duration[19]. After surgical cure of endogenous CS, GWS is usually characterized by biochemical evidence of hypothalamic-pituitary-adrenal (HPA) axis suppression with many signs and symptoms consistent with cortisol deficiency despite the use of supraphysiologic glucocorticoid replacement therapy. The degree of physical or psychologic glucocorticoid dependence experienced by patients may not correlate with the degree of HPA axis suppression[20, 21].

**Fig. 1:**
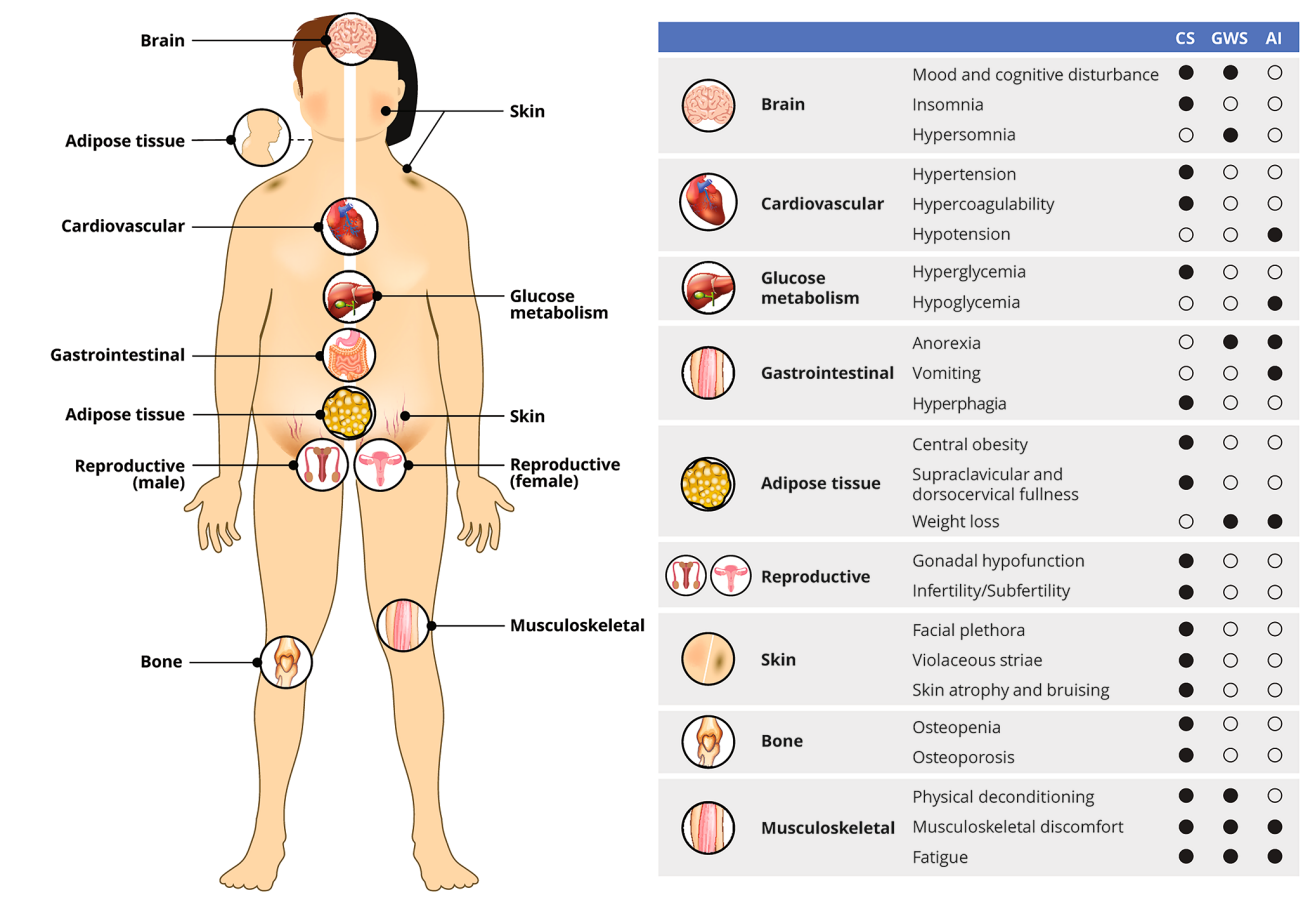
Overlapping clinical features of Cushing syndrome (CS), glucocorticoid withdrawal syndrome (GWS), and adrenal insufficiency (AI)

GWS symptom onset is typically 3–10 days postoperatively, often after the patient has been discharged from the hospital. The first symptoms of GWS vary but usually consist of myalgias, muscle weakness, fatigue, and hypersomnolence. Anorexia, nausea, and abdominal discomfort are common, but vomiting should raise concern for hyponatremia, cerebrospinal fluid leak, hydrocephalus, or other perioperative complications. Mood changes develop more gradually and range from mood swings to depression, and the fatigue with myalgias can exacerbate mood changes. An atypical depressive disorder has been described in many patients after CD surgery[22]. Weight loss should ensue in most patients but gradually and proportionate to the reduction in glucocorticoid exposure. It is important to complete a thorough symptom review and physical exam at postoperative visits, as the differentiation between GWS and bona fide AI – and even between GWS and recurrence of CS – can be challenging (Fig. 1). All three conditions are associated with symptoms of myalgias, weakness, and fatigue; however, rapid weight loss, hypoglycemia, and hypotension are suggestive of AI and the need for an increase in the glucocorticoid dose. In parallel, hypersomnia is more suggestive of GWS, while insomnia is more associated with recurrence of CS. Given the anticipation of GWS onset shortly after discharge and the potential for hyponatremia during this time, a widely employed strategy is a generous glucocorticoid dose for the first 2–3 weeks, at least until the first postoperative outpatient visit (Table 1).

**Table 1:** Glucocorticoid Therapy Options After Surgery for CS

Medication	Initial Total Daily Dose	Tapering Increment	Comments
Hydrocortisone	30–60 mg/day	5–10 mg/day	Shift to circadian rhythm in 2–6 weeks
Prednisone	10–20 mg/day	1–2 mg/day	Pro-drug, unreliable
Prednisolone	10–20 mg/day	1–2 mg/day	
Methylprednisolone	8–16 mg/day	1–2 mg/day	
Dexamethasone	2–5 mg/day	0.25–0.5 mg/day	Difficult to titrate once daily dose < 1 mg/day
Fludrocortisone acetate	0.1–0.4 mg/day	0.05–0.1 mg/day	Only after BLA

BLA, bilateral adrenalectomy

The mechanisms responsible for the precipitation of the GWS after surgery for CS and the variability in its manifestations are not completely understood, yet alterations in the regulation of cortisol and cortisol-responsive genes appear to contribute. Down-regulation of corticotropin-releasing hormone (CRH) and proopiomelanocortin (POMC) expression, combined with up-regulation of cytokines and prostaglandins are likely to be important components of GWS. Low CRH has been associated with atypical depression[23], and CRH levels in cerebrospinal fluid of patients with CD are significantly lower compared to healthy subjects[24]. CRH suppression gradually resolves after surgical cure over 12 months during glucocorticoid replacement[25], illustrative of the slow recovery process. The expression of POMC, the ACTH precursor molecule, is also suppressed with chronic glucocorticoid exposure[26], and the normalization of POMC-associated peptides mirrors HPA axis recovery[19]. In the acute phase of glucocorticoid withdrawal, interleukins IL-6 and IL-1β, as well as tumor-necrosis factor alpha (TNFα) have been observed to rise[27], suggesting that glucocorticoid-mediated suppression of cytokines and prostaglandins is then released in GWS, and these cytokines induce the associated flu-like symptoms. Glucocorticoid replacement with dexamethasone 0.5 mg/d reduced but did not normalize IL-6 after 4–5 days[27], consistent with resistance to suppression during GWS.

## Acute Care: Perioperative Planning, Coaching, and Management

For patients with CD, transsphenoidal surgery performed by an experienced surgeon achieves remission in about 80% of pituitary microadenomas and 60% of macroadenomas[28–31]. Post-operative AI and GWS are some of the most challenging phases of management for endocrinologists and one of the most disheartening for CS patients. Many patients report feeling unprepared for the postsurgical recovery process[32]. *For these reasons, it is important to prepare the patient prior to surgery for the difficult months ahead, and the same considerations apply to the commencement of medical therapies, as will be discussed later.* On the one hand, more potent glucocorticoids and higher doses reliably mitigate symptoms, but on the other hand, substitution of exogenous for endogenous CS delays recovery of the HPA axis and perpetuates CS-related co-morbidities. Limited data that compare management strategies preclude evidence-based decisions, yet some themes can be derived from expert opinion and extensive experience from CS centers.

In centers dedicated to the management of CS, surgeons and endocrinologists work closely together through all phases of the process. Although the goal of primary surgery for CD is adenoma resection, the tumor might not be found and/or removed completely after initial exploration. To prepare for this possibility, the surgeon should determine in advance with the patient and endocrinologist what to do next in this situation – dissect further, perform a hypophysectomy or hemi-hypophysectomy, or stop the operation. The plan for perioperative testing and glucocorticoid treatment varies widely among centers. The conundrum faced in the immediate perioperative period is that withholding glucocorticoids allows for rapid testing and demonstration of remission; however, complete resection of the causative tumor causes AI from prolonged suppression of the HPA axis and concerns for acute decompensation. Abundant evidence has shown that post-pituitary adenomectomy patients are not at risk for an adrenal crisis when monitored closely in an intensive care unit or equivalent setting[33]. Many studies have confirmed that post-operative AI almost always suggests a remission of CD[15–18, 34]. A standard protocol includes securing serum electrolytes and cortisol, plasma ACTH, capillary blood glucose, blood pressure, and urine specific gravity every 6 h for 24–48 h while withholding all glucocorticoids. Consecutive serum cortisol values less than 2–5 µg/dL (we use < 3 µg/dL) are sufficient to document successful tumor resection and to begin glucocorticoid therapy[35]. Post-operative signs and symptoms of AI including vomiting, hyponatremia, hypoglycemia, and hypotension should also mandate immediate glucocorticoid support. Although not clinically useful in the immediate post-operative period, some investigators have shown that low ACTH and DHEAS levels may be better predictors of long-term remission than serum cortisol[36]. A similar strategy for the management of possible post-operative AI/GWS following unilateral adrenalectomy for nodular adrenal disease has recently been reported. A post-operative day 1 basal cortisol and its response to cosyntropin stimulation can reliably segregate those patients with HPA axis suppression requiring cortisol replacement from those with an intact HPA axis who do not need to be discharged with glucocorticoid therapy[37].

Once remission is achieved, exogenous glucocorticoid replacement should be initiated and maintained during the months required for HPA axis recovery. Several glucocorticoids and dosing options are available (Table 1), and the initial dose is generally 3- to 4-fold higher than the physiologic range and graded based on age, comorbidities, and severity of disease. Fludrocortisone acetate should also be initiated following BLA for patients who receive glucocorticoids other than hydrocortisone, the only glucocorticoid with mineralocorticoid activity. By comparison, post-BLA patients receiving supraphysiologic hydrocortisone doses usually do not need mineralocorticoid support until their dose is tapered to near physiologic replacement. In the acute postoperative period, several medical comorbidities accompanying CS may reverse rapidly and require medication adjustments[35]. In particular, insulin and oral hypoglycemic drugs, potassium-sparing diuretics such as spironolactone, and other cardiovascular drugs are typically tapered or discontinued as glucose counter-regulation and electrolyte balance change rapidly upon cortisol reduction. Due to the high risk of postoperative venous thromboembolism[38–40], prophylaxis is frequently recommended and continued for several weeks after discharge. Posterior pituitary manipulation can disturb water balance and result in serum sodium alterations, including transient or permanent central diabetes insipidus, and in rare cases the triphasic response of diabetes insipidus, followed by syndrome of inappropriate secretion of antidiuretic hormone (SIADH), and finally permanent diabetes insipidus[41, 42]. In the first week or two after discharge, the most common cause for readmission is hyponatremia[43, 44], although the mechanisms responsible for this transient SIADH state are not known. For this reason, patients should be instructed to drink only when thirsty and not as an alternative to solid foods or for social reasons for 7–10 days after the surgery. Both diabetes insipidus and SIADH may not manifest for weeks after surgery; consequently, serum sodium should be monitored after hospital discharge as well [42].

## Subacute Care: The GWS and HPA Axis Recovery

When managing GWS symptoms, it is important to repeatedly emphasize to the patient that not only are GWS symptoms to be expected, but in fact these manifestations portend a favorable prognosis of sustained remission from CS. The most important treatment intervention is frequent reassurance to the patient that GWS typically resolves as the HPA axis recovers. Family members must be included in the conversation to help provide as much support as possible, as patients report that support from family and friends is the most helpful coping mechanism during the recovery process[32]. When appropriate, it may be necessary to provide the patient with temporary disability documentation, since GWS symptoms may be so severe to preclude gainful employment. The patient must know that the myalgias reflect the body’s attempts to repair the muscle damage, similar to the soreness experienced the day after resistance weight training, and these aches will eventually subside. Due to the challenges of differentiating between GWS and AI, a higher glucocorticoid dose can be briefly trialed to assess if this increased glucocorticoid exposure improves symptoms, but late-day dosing should be avoided to support recovery of the circadian rhythm. In parallel, the patient should be encouraged to adequately rest, particularly going to sleep early but limiting daytime sleep to short naps.

Several other classes of medications can be trialed to target specific patient symptoms (Table 2). Antidepressants such as fluoxetine, sertraline, and trazodone might help to improve mood, sleep and appetite. A non-steroidal anti-inflammatory medication to address the musculoskeletal discomfort might be used early in the GWS, with the cyclooxygenase type 2 (COX-2) inhibitor celecoxib (100–200 mg once or twice daily) preferred when several weeks of daily treatment is needed, generally not more than 3 months. With anorexia and reduced food intake, adequate protein intake is necessary to allow muscle recovery. Egg whites, nuts, and lean meats are nutritionally dense and generally easy to tolerate despite poor appetite.

**Table 2:** Pharmacotherapy and Ancillary Treatment Options for GWS Symptoms

Medication / Modality	Examples	Signs / Symptoms Addressed
Antidepressants	Sertraline, fluoxetine, trazodone	Mood disturbance, anorexia, poor sleep
Nonsteroidal anti-inflammatory drugs	Celecoxib	Musculoskeletal discomfort
Bone-directed drugs	Alendronate, zoledronate, teriparatide, abaloparatide	Osteoporosis, fragility fractures
Physical therapy		Physical deconditioning, musculoskeletal discomfort
Cognitive therapy		Mood disturbance, disease coping

Following surgical remission, the duration of glucocorticoid taper can vary from 6 to 12 months or more, depending on age, severity of disease, and duration of disease [45, 46]. Monitoring for HPA axis recovery involves both clinical and biochemical assessments. Since the HPA axis is likely to remain suppressed with prolonged supraphysiologic glucocorticoid replacement, the first goal is to shift from all-day dosing to a circadian schedule as soon as possible, such as hydrocortisone 20 mg on rising and 10 mg in the early afternoon by 2–6 weeks after surgery. The advantages of hydrocortisone include rapid absorption for symptom mitigation, the ability to measure serum cortisol as a measure of drug exposure when helpful, and the relatively short half-life [47], which ensures a glucocorticoid-free period in the early morning when it is most critical to avoid prolonged HPA axis suppression and to enhance recovery. The second goal, which should not be attempted until GWS symptoms – particularly the anorexia and myalgias – are considerably improved, is to limit replacement to a single morning dose.

**Fig. 2:**
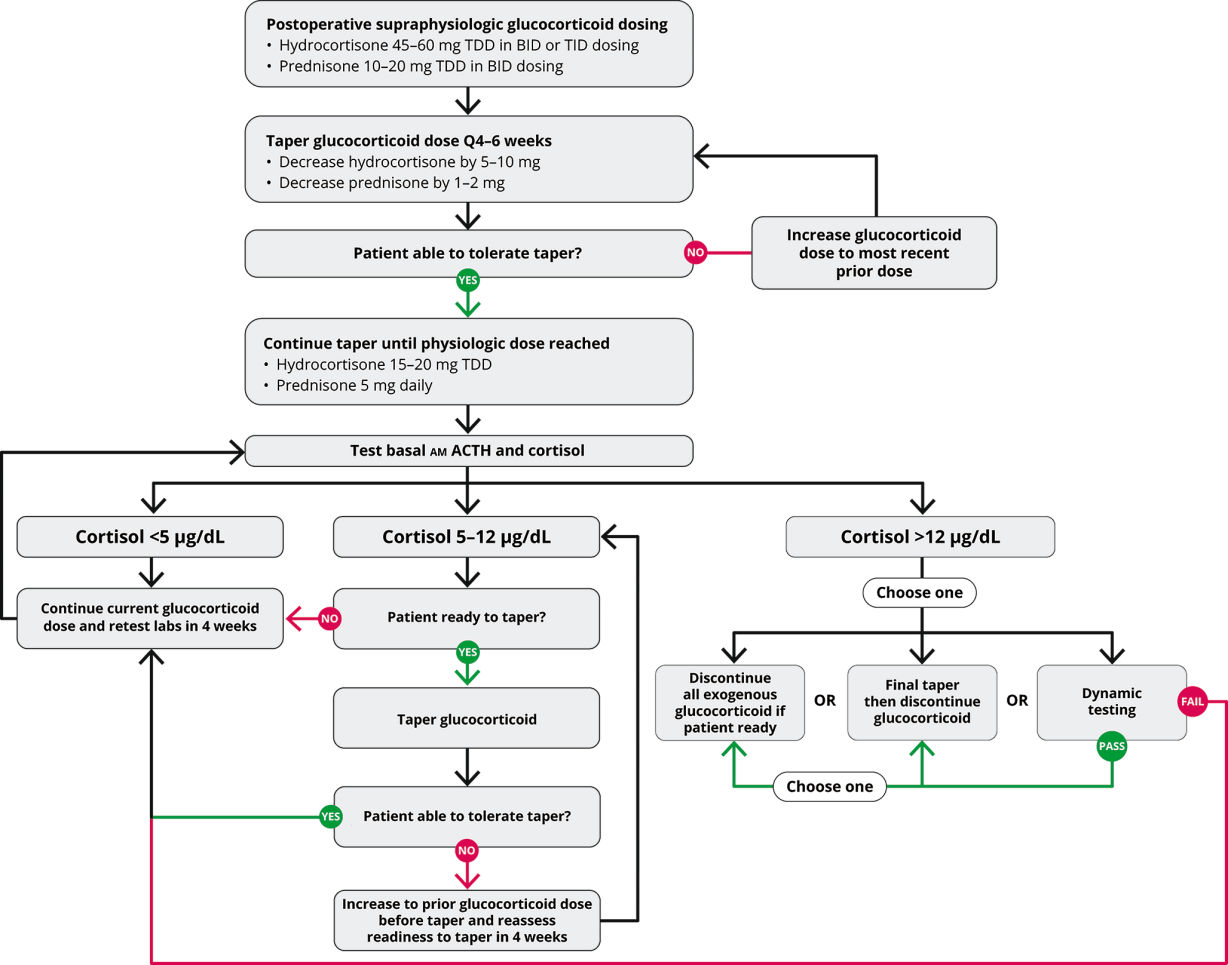
Glucocorticoid withdrawal algorithm. TDD, total daily dose

Biochemical assessment should begin once patients are taking a physiologic dose of glucocorticoid replacement (total daily dose of hydrocortisone 15 to 20 mg per day) and clinically feel well enough to begin the final stage to discontinuation of glucocorticoid replacement (Fig. 2). Biochemical evaluation begins with basal testing, and dynamic assessment of adrenal function might be necessary to confirm completion of recovery. For basal testing, patients should not take their afternoon hydrocortisone dose (if prescribed) the day before testing and then have a blood draw by 0830 prior to the morning hydrocortisone dose on the day of testing. While a serum cortisol alone is adequate to taper hydrocortisone, a simultaneous plasma ACTH assists in gauging the state of HPA axis recovery. Often the ACTH and cortisol rise gradually in parallel, but sometimes the ACTH rises above the normal range despite a low cortisol, which indicates recovery of the hypothalamus (CRH neuron) and pituitary corticotrophs in advance of adrenal function. Serum DHEAS can remain suppressed for months to years after cortisol normalization, and a low DHEAS does not indicate continued HPA axis suppression. A rapid rise in DHEAS, in contrast, is concerning for disease recurrence, but a slow drift to a measurable amount in parallel with the cortisol rise is consistent with HPA axis recovery. Periodic assessment of electrolytes is prudent to screen for hyponatremia and hypo- or hyperkalemia as medications are changed, particularly diuretics. Hypercalcemia that is parathyroid-hormone independent might be observed during the recovery phase, probably related to the rise in cytokines that accompany resolution of hypercortisolemia[48, 49].

Basal testing is performed at 4- to 6-week intervals during glucocorticoid replacement. A rule of thumb is that the AM cortisol in µg/dL plus the morning dose of hydrocortisone in milligrams should sum to 15–20. Thus, once endogenous cortisol production is measurable, the hydrocortisone dose should be not more than 20 mg on arising. Once the AM cortisol rises to near 5 and then 10 µg/dL, the AM hydrocortisone dose is dropped to 15 and then 10 mg, respectively. Once the AM cortisol is 12–14 µg/dL, recovery is essentially complete, and the morning hydrocortisone dose is dropped to 5 mg for 4–6 weeks and then stopped or held for dynamic testing (Fig. 2). A clinical pearl related to HPA axis recovery is that patients who state that they are finally feeling better and getting over the GWS usually have started to make some endogenous cortisol, yet not enough to stop glucocorticoid tapering. Nevertheless, a smidgeon of endogenous cortisol production with the waning of GWS symptoms is a harbinger that HPA axis recovery is imminent. If basal testing is equivocal, dynamic testing might be necessary. The gold standard testing for central AI is the insulin tolerance test, which is rarely used, and metyrapone testing might be employed once the basal cortisol is > 10 µg/dL. Although designed to test for primary adrenal insufficiency, the cosyntropin stimulation test is often employed in this setting due to greater availability, simplicity, and safety than insulin or metyrapone testing. The duration of full HPA axis recovery can be highly variable depending on the individual and postoperative glucocorticoid dosing[50].

## GWS During Medical Management of CS

Patients who are not surgical candidates or do not have successful remission of CS following surgery may be offered medical treatment or BLA. After BLA, the GWS will ensue without eventual recovery of the HPA axis, so glucocorticoids are tapered until a chronic physiologic replacement dose is reached as described previously. With medical management, patients might also experience GWS, particularly at the onset of treatment. Therefore, patients must be counseled that the typical symptoms of fatigue, myalgias, and anorexia are not only possible but indeed expected, rather than “side effects” of the medication, with two caveats. First, as described for glucocorticoid replacement following surgical remission, the endocrinologist must distinguish GWS from AI due to over-treatment of CS. The same parameters of vomiting, hypotension, and hypoglycemia favor inadequate cortisol exposure and the need for dose reduction or treatment pause and/or supplementation with a potent glucocorticoid such as dexamethasone to reverse an acute event. Second, known adverse effects of the specific drug in use should be considered and excluded. The quandary of distinguishing GWS from over-treatment raises an important principle of medical management: under-dose initially and gauge primarily the severity of GWS symptoms in the first several days. The initial goal of medical therapy is not to rapidly achieve normal cortisol milieu, but rather to “dial in” just enough inhibition of cortisol production or receptor antagonism to precipitate mild to moderate GWS symptoms. Once GWS symptoms appear and/or a typical dose of the medication is achieved, further assessments, including glucose, serum cortisol and/or UFC (except when treated with mifepristone), clinical appearance, and body weight are conducted while the dose is maintained constant until GWS symptoms begin to dissipate. If the patient is not experiencing adequate clinical and/or biochemical benefit from the medication in the absence of GWS symptoms, the dose is gradually raised incrementally. This iterative process might require periodic dose reduction or perhaps even temporarily discontinuing the medication if the patient’s daily living activities are affected at any point in the process.

For several medications, a block-and-replacement strategy is an option[3], particularly for very compliant patients for whom a priority is placed on avoidance of over-treatment. This strategy resembles thionamide-plus-levothyroxine therapy for the treatment of Graves disease. The patient is given both a generous dose of medication to completely block endogenous glucocorticoid production, plus simultaneous exogenous glucocorticoid therapy, titrated to replacement dose or greater. This approach allows for greater control over glucocorticoid exposure and low risk of AI, as long as the patient always takes both medications each day. Long-acting pasireotide, for example, would not be an appropriate drug for the block-and-replace strategy. Based on the drug mechanism of action, this block-and-replace strategy is feasible with ketoconazole or levoketoconazole, the 11β-hydroxylase inhibitors osilodrostat and metyrapone, and the adrenolytic agent mitotane (the latter three are off-label uses). Alternatively, the patient might be given a double replacement dose of glucocorticoid to take only if symptoms concerning for over-treatment occur, and the medical therapy for hypercortisolemia is then interrupted until the patient communicates with the endocrinologist.

Treatment monitoring with medical management includes biochemical and symptom assessment. For all medications other than mifepristone, normalization of 24-hour UFC is the minimal goal [2]. Basal morning cortisol and late-night salivary cortisol may be more challenging to interpret in the setting of diurnal rhythm loss characteristic of CS. Because mifepristone blocks glucocorticoid receptors, ACTH and cortisol increase with treatment for most forms of CS; dose titration therefore relies on assessment of clinical features, glycemia, body weight, and other metabolic parameters [2]. For occult tumors, periodic imaging to screen for a surgical target and/or tumor regrowth is prudent, and a pause in treatment for repeat surgery might be indicated.

## The End Game: Comprehensive Recovery for the Patient with CS

Besides navigating the GWS and shepherding recovery of the HPA axis, recovery from co-morbidities of CS must be addressed to the extent possible. Hypertension, hyperglycemia, hypokalemia, and dyslipidemia often improve substantially but do not always resolve. Insomnia, skin thinning and bruising, and risk of thrombosis also generally resolve, and associated treatments might be discontinued. Although there is usually an improvement in bone density and decreased fracture risk following correction of CS, anabolic and/or anti-resorptive therapies may be warranted in some patients. The deformities of vertebral compression fractures may be permanent, and some authors have recommended the use of vertebroplasty for symptom relief[51]. Violaceous striae and chronic skin tears might heal with hyperpigmentation, leaving “the scars of Cushing’s,” which can persist for a lifetime. These milestones or minor victories can be used as evidence of healing and encouragement for the patient during the dark days of the GWS, and these changes herald further improvements. Fat redistribution and significant weight loss take some weeks to manifest and usually follow next.

The myopathy from CS is an example of a co-morbidity that rarely improves without targeted treatment, and the German Cushing’s Registry has provided evidence for chronic muscle dysfunction following cure of CS[52]. Recent data indicate that a low IGF-1 after curative surgery is associated with long-term myopathy [53]. This persistent myopathy is a common source of chronic fatigue following HPA axis recovery, which is unresponsive to glucocorticoids. For these reasons, an important ancillary modality is physical therapy, and an ideal time to initiate this treatment is at the first signs of HPA axis recovery when the GWS symptoms have subsided. A complete evaluation from an experienced physical therapist should focus on core and proximal muscle strength, balance, and other factors that limit function. Exercises targeting these factors (stand on one foot, sit-to-stand, straight-arm raises with 1- to 5-pound weights) rather than traditional gym exercises (arm curls, bench press, treadmill) are necessary to restore functional status and avoid frustration and injury when the patient is not yet prepared for the latter stages of recovery. Professional supervision of this initial phase is a critical component of the recovery process, and failure to attend to musculoskeletal rehabilitation – as would be routine following survival of a critical illness – risks long-term morbidities from a curable disease.

Patients with CS often complain of cognitive defects, which usually improve but may not completely recover following treatment[54, 55]. Glucocorticoids are toxic to the hippocampus, and both rats treated with high-dose corticosterone and patients with CD experience reductions in hippocampal volume, which does not completely return to normal even with correction of hypercortisolemia[56, 57]. Because the hippocampus is an important brain region for memory, the main complaint is impaired formation of new memories and recall of recent events. When significant cognitive dysfunction persists, a formal neuropsychologic testing session is prudent, both to screen for additional sources of memory loss (degenerative brain diseases) and to identify aspects that might be amenable to functional management approaches. Cognitive therapy can be effective for mental health and overall disease coping strategies as well.

Finally, for patients undergoing transsphenoidal surgery for CD, complications associated with pituitary surgeries in general should also be considered. Anterior pituitary hormone axes should be assessed biochemically and symptomatically for hypothyroidism and hypogonadism, as hypopituitarism is an independent predictor of decreased quality of life after surgical cure [58]. Hypopituitarism can not only complicate the assessment of GWS with overlapping symptoms such as fatigue, but treatment of hypopituitarism can also be important for GWS recovery. Prior to initiating physical therapy, testosterone replacement in male patients with hypogonadism should be optimized. Hypothyroidism can contribute to hyponatremia and can also slow the metabolism of glucocorticoids. Therefore, optimizing the treatment of hypothyroidism and hypogonadism prior to completing glucocorticoid taper is prudent. Growth hormone deficiency may also be evaluated in symptomatic patients in the setting of other anterior pituitary hormone deficiencies, although formal evaluation is best delayed for at least 6–12 months when HPA axis recovery has occurred or at least the glucocorticoid dose is reduced to a physiologic range [2].

## Summary and Final Thoughts

After a diagnosis of CS has been well established, a multidisciplinary team of endocrinologists and surgeons must design the best treatment strategy for the patient. Expectations and possible adverse side effects of surgery or pharmacotherapy should be reviewed with the patient. The GWS is a very difficult concept for patients to understand. It seems inconceivable to them that they could possibly feel worse (and that this is a good omen) six weeks after resolution of their hypercortisolism than they do pre-operatively; however, there are no studies that address whether comprehensive pre-operative patient education regarding GWS has any impact on the patient’s post-operative perception and outcome after successful surgery. An addiction metaphor is sometimes helpful: the patient’s body and brain has become addicted to steroids (cortisol) and after steroids are abruptly reduced, their body and brain are dysphoric — much like removal of any other addictive substance (e.g., opioids, alcohol, nicotine). The patient and their care team need to know that this treatment odyssey will be a marathon, not a sprint. It may take as long as 12–18 months for patients to have full HPA axis recovery, regression of GWS, and, most importantly, resolution of the devastating effects of chronic excessive glucocorticoid exposure.

## Conclusions

GWS following surgery or during medical treatment of CS can be challenging to manage. There are currently no standard guidelines for management of GWS, but various available medical and ancillary therapies are discussed here. Studies are needed to better understand the pathophysiology of GWS to guide more targeted treatments. There may be yet unrecognized steroids produced by the adrenal glands, the withdrawal of which contributes to GWS symptoms[59]. Future observational and interventional studies would be beneficial for identifying optimal management options.

## Electronic Supplementary Material

Below is the link to the electronic supplementary material.

Supplementary Material 1
